# Arduino-Based Readout Electronics for Nuclear and Particle Physics

**DOI:** 10.3390/s24092935

**Published:** 2024-05-05

**Authors:** Markus Köhli, Jannis Weimar, Simon Schmidt, Fabian P. Schmidt, Alexander Lambertz, Laura Weber, Jochen Kaminski, Ulrich Schmidt

**Affiliations:** 1 Physikalisches Institut, Heidelberg University, Im Neuenheimer Feld 226, 69120 Heidelberg, Germany; 2 Physikalisches Institut, University of Bonn, Kreuzbergweg 24, 53115 Bonn, Germany

**Keywords:** open hardware, radiation detection, particle physics, proportional counter, SiPM readout

## Abstract

Open Hardware-based microcontrollers, especially the Arduino platform, have become a comparably easy-to-use tool for rapid prototyping and implementing creative solutions. Such devices in combination with dedicated front-end electronics can offer low-cost alternatives for student projects, slow control and independently operating small-scale instrumentation. The capabilities can be extended to data taking and signal analysis at mid-level rates. Two detector realizations are presented, which cover the readouts of proportional counter tubes and of scintillators or wavelength-shifting fibers with silicon photomultipliers (SiPMs). The SiPMTrigger realizes a small-scale design for coincidence readout of SiPMs as a trigger or veto detector. It consists of a custom mixed signal front-end board featuring signal amplification, discrimination and a coincidence unit for rates of up to 200 kHz. The nCatcher transforms an Arduino Nano to a proportional counter readout with pulse shape analysis: time over threshold measurement and a 10-bit analog-to-digital converter for pulse heights. The device is suitable for low-to-medium-rate environments up to 5 kHz, where a good signal-to-noise ratio is crucial. We showcase the monitoring of thermal neutrons. For data taking and slow control, a logger board is presented that features an SD card and GSM/LoRa interface.

## 1. Introduction

Complementary to the scaling of high-performance computing architecture, low-cost mini-computers and stripped-down microcontrollers are increasingly populating researcher’s toolboxes [1]. They often replace costly dedicated table-top or rack-mounted devices. Opening new possibilities, some even provide the basis for an entire measurement setup as the examples of MuonPi [2] and CosmicPi [3] showcase. With the Arduino Open-Hardware electronics platform [4], microcontrollers (MCUs) have become a comparably easy-to-use tool for rapid prototyping [5], and they have provided grounds for a wide range of open-source solutions [6]. Their modularity qualifies them as a teaching platform [7,8], and they made their way into research labs [9] in experiments as part of the front-end [10,11], the back-end [12,13] and automation control [14,15], as well as multi-level machine interfaces [16,17], specifically the Internet-of-Things [18,19]. With the upsurge of miniaturized sensor technologies, their interface capabilities provide direct and easy solutions [20] for monitoring platforms [21,22] - particularly in the case of independently operating systems with limitations on power consumption as is often the case for environmental sciences [23,24,25] and nuclear science [26,27,28]. Compared to other microcontrollers, the community around Arduino system is extensive, which facilitates building custom solutions. In particular, their stability, wide voltage and temperature range, hardware bus interfaces and low power consumption qualifies them for slow control units. Yet, running at frequencies of several MHz, their capabilities can be extended to data taking and signal analysis at mid-level rates. Such devices in combination with dedicated front-end electronics can offer low-cost alternatives for student projects and independently operating small-scale instrumentation.

We present two projects for detecting charged particles and photons, which realize the readout of proportional counters for neutron detection and silicon photomultipliers coupled to scintillators or wavelength-shifting fibers. Earlier versions of the projects have contributed to the detectors described in [29,30].

## 2. Materials and Methods

**Figure 1:**
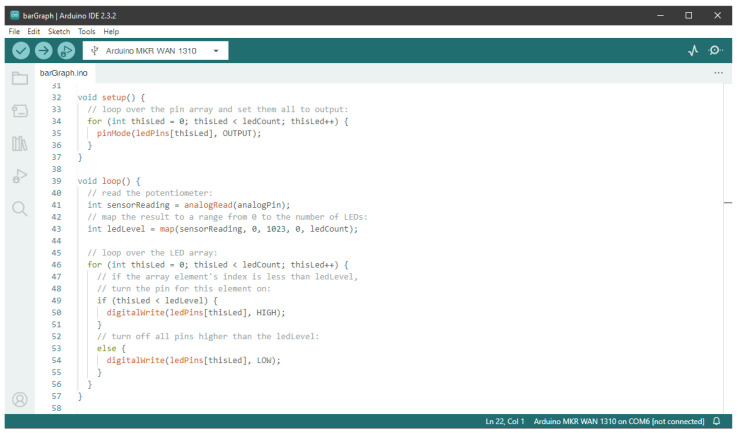
Arduino IDE with a C++ example showing the buildup of a simple program. The microcontroller first initializes itself in the setup() routine, and then infinitely runs the loop(). The internal analog-to-digital converter measures the voltage level on an input pin using the library function analogRead, and then uses digital output pins as status indicators.

The Arduino platform, originally designed for a general audience, has become a versatile tool for researchers in recent years, especially for those who want to realize laboratory setups quickly and cost effectively. The term Arduino refers to a series of microcontroller boards of different complexities with focuses on interoperability and directly accessible user interfaces. Without additional hardware, the user can program and interact with each device through the Arduino library and drivers over a standard USB connection [31]. The success of this platform, therefore, is based on several features that set it apart from other microcontroller platforms, such as the following:

Simplicity and ease of use: It was developed with the aim of making it easy for beginners to get started with microcontroller programming. The development environment (IDE) (see Figure 1) is comparably easy to use and offers a wide range of examples that allow beginners to start developments quickly.
Open-source code: The Arduino platform is based on open-source software and hardware. This means that the entire hardware specification is freely available, and the libraries and drivers are supported by an active developer community. Researchers can easily customize and extend solutions to suit their needs.
Large ecosystem of add-ons: Arduino microcontrollers have a broad range of available expansion boards, so-called shields, sensors, actuators and other extensions. This allows researchers to quickly develop prototypes and add functions without having to develop each component individually. Built-in hardware support for common interfaces allow researchers either to directly add a considerable set of professional components or to address interface adapter chips to bridge a larger range of protocols.
Cost-effectiveness: Most solutions for Arduino boards, including plug-in hardware, are more cost effective than advanced devices and simpler compared to many other microcontroller platforms. This allows researchers with limited budgets to conduct extensive experiments and develop prototypes without large investments upfront.
Flexibility: Arduino-based solutions can be used for a wide range of applications, from simple teaching solutions to table-top experiments or complex automation and control systems. The platform supports a variety of programming languages and can be used with different operating systems or integrated into professional IDEs.
Collective expertise: With a crowd-sourced knowledge pool, the key expertise for a setup is not bound to one or a few persons. Projects can be transmitted comparably easy between different students or groups with a high fluctuation of members.

An Arduino board has a set of digital pins that can be configured as input or output with some of them also allowing Pulse Width Modulation (PWM). Furthermore, the board is provided with analog inputs that can be fed to a 10-bit analog-to-digital-converter (1024 different values), a crystal oscillator, a USB connection, a power jack and an In-Circuit Serial Programming header (ICSP). Typically an Arduino can be powered using a USB or with an external power supply, for example, a 9-V battery or a 12-V-DC-power supply. On small microcontrollers, the memory is limited. The ATmega328 [32], for example, has just 32 KB, with 2 KB used for the bootloader (it can be reduced to 0.5 KB using Optiboot [33]). It also has 2 KB of static RAM and 1 KB of EEPROM [34], which can be read and written with the EEPROM library. Depending on the microcontroller, I/O operates at 5 V or 3.3 V. Each pin can provide or receive a maximum of 40 mA and has an optional internal pull-up resistor of (1–50) kΩ. Some inputs can be configured to trigger an interrupt on a low value, a rising or falling edge. In addition, some pins have specialized interface functions [35]: pairs of pins may be used to receive (RX) and transmit (TX) serial data [36] up to 115,200 Baud, $I^{2}$C commands [37] through a two-wire interface with clock (SCK) and data (SDA) at 100 kHz or 400 kHz and. finally, a four-wire SPI [38] transmission at up to 8 MHz on most Arduinos or 48 MHz on an Arduino DUE [39]. Table 1 provides an overview of the microcontrollers used in this project and additionally lists upgrade options for advanced STM32 platforms. Typical shortcomings for laboratory use are the lack of an onboard real-time clock (RTC) and a comparably simple memory management, which impede the use of high-level structures in programming for more complex solutions.

## 3. System Design

**Figure 2:**
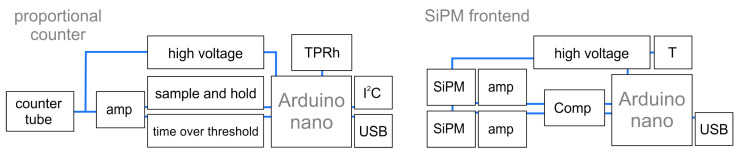
Schematic design of both types of detector front-end electronics with the main functional components for one channel. Whereas the proportional counter readout (**left**) measures pulse length and pulse height, the SiPM board (**right**) sets a fixed comparator threshold and counts coincidences between both photon counters. Additionally, both designs include sensors for environmental variables like temperature *T*, pressure *p* and relative humidity Rh.

Proportional counters [42] or detectors based on scintillating materials [43] are widely used to detect ionizing radiation or neutral particles through conversion processes [44]. These are the most fundamental detection types used to quantitatively measure particles and their energy. With such detectors being deployed in most experiments in nuclear and particle physics and developed to maximize their resolution and rate capability, we focused on realizing a basic but functional concept behind the frontier high-performance hardware, yet using state-of-the-art off-the-shelf components. Our designs, shown in Figure 2, rely on using the Arduino microcontroller as a digitizer and slow-control interface, whereas the analog stage and voltage supply are part of our external board design.

### 3.1. Board Design

**Figure 3:**
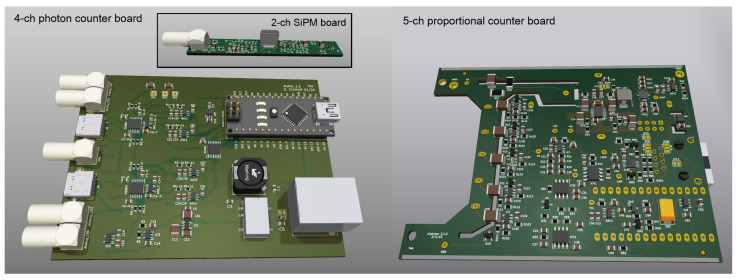
Three-dimensional rendering of both Arduino-based readout boards. The photon counter front-end (left) is split into a dedicated amplifier board (inlet) with short signal lines to the SiPMs and a digitizer for the coincidence counting of two two-channel boards. The proportional counter front-end features up to five channels, which are fed into one sample, and holds the comparator circuit. Both boards are equipped with a high-voltage module on their backside. The Arduino Nano can be plugged into the corresponding socket row as shown on the left.

The printed circuit board (PCB) (see Figure 3) influences the performance of electronic circuits, including the component placement, traces and planes. A crucial prerequisite to low-noise performance is a well-designed layout of the PCB. The digital part can induce electromagnetic interference (EMI) on the analog part. Digital and analog signal separation, therefore, is critical in mixed systems. The ±5 V analog voltage supply is generated in the vicinity of its consuming integrated circuits (ICs) with low-noise linear regulators. There is no ground plane below the integrator input stage in order to minimize capacitive coupling. Otherwise, large ground and voltage planes help to reduce the noise. Analog signals are transmitted through slightly larger traces on one side of the PCB only in order to reduce their impedance. Several high- and low-pass filter stages significantly increase the signal-to-noise ratio.

The comparator IC links the analog circuitry to the digital part through star point grounding. The high-voltage traces are also followed by a ground plane below, which leads to an electric field that points downward into the PCB rather than pointing in the direction of the signal lines. Additionally, longitudinal mill traces between the high voltage and analog part impede charge movement across the board. For analog signal transmission, we used USB-C connectors, which feature a comparably high voltage protection and good signal insulation. Optionally, signal paths can be readout externally using Lemo 00 series connectors. Digital signals and power lines are realized over one RJ45 socket.

### 3.2. Proportional Counter Readout nCatcher

Counting tubes are mostly designed as hermetically sealed cylinders with a thin wire in their center. A high voltage is applied to the wire, which creates a radially symmetric electrical field that transports electrons to the anode wire, where avalanche formation takes place, and the residual ions are then transported to the cylinder walls.

The high voltage applied to the wire is chosen in such a way that the number of electrons generated within the charge avalanche is increased by a constant gain factor. Hence, the number of electrons collected at the wire is proportional to the number of primarily generated electrons, which are in turn proportional to the energy deposited by the charged particle. This relationship between the energy deposition of a charged particle and the number of electrons is the key characteristic of a proportional counter.

**Figure 4:**
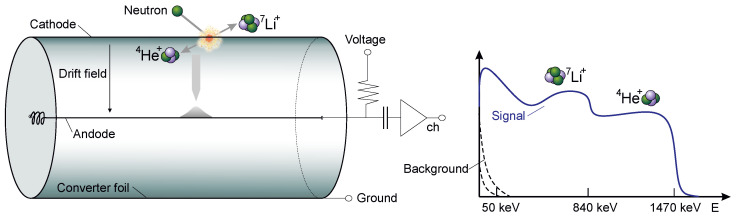
Schematic of a proportional counter for detecting neutrons, which are converted into ions, emitted back-to-back, by a coating on the counter wall. An electric field between the tube wall and the axial wire accelerates the generated electrons towards the center. In the vicinity of the wire, the electrons reach the gas ionization energy threshold and charge multiplication takes place. The pulse is then read out by a charge sensitive amplifier. The resulting spectrum is shown on the right graph. From the maximum energy deposition of the nuclear fragments the spectrum is extended downwards due to losses in the converter medium itself. For in this case overlapping background noise a signal threshold is required.

The key feature of any proportional counter, therefore, is to precisely measure the charge generated in the ionization process by the incoming particle. With a well-adapted analog amplifier stage, one can distinguish between the dense tracks from ions and weaker ionization processes from lighter particles like muons, electrons and protons. Energy discrimination can also effectively suppress most ionization events from gammas. However, some of these particles can deposit significant amounts of energy in the gas if their track length is large enough. Long ionization traces lead to significant differences in the arrival of the primary electrons close to the counter wall and those close to the wire. In general, the projected axial ionization path length directly relates to the drift time of the charge pulse [45,46]. As opposed to electrons, the rise time generated by ions is very short due to the short-ranged and dense ionization processes. Pulse rise time is another tool for particle discrimination [47]. The design goal for our readout electronics was, therefore, to precisely determine the height and rise time of the charge pulse, both quantities related to the energy loss per unit path length [48]. Moreover, the demands for such detectors are insensitive to temperature drifts, air humidity and low background noise induced by the electronic circuit itself. In the here presented case of a neutron detector, proportional counter tubes are read out, see Figure 4, with boron-10 being applied as a converter by the using process

$$\begin{array}{ccc} \; & {\;}^{10}B+n\to & \\ \; & {\;}^{7}\mathrm{Li}(0.84\;\mathrm{MeV})+\alpha (1.472\;\mathrm{MeV})+\gamma (0.48\;\mathrm{MeV}) & (93.6\;\%),\\ \; & {\;}^{7}\mathrm{Li}(1.013\;\mathrm{MeV})+\alpha (1.776\;\mathrm{MeV}) & (6.4\;\%). \end{array}$$

With the inner walls of the counter tube being covered by sputter-deposited ${\;}^{10}B_{4}$C, see also [49,50], the partial energy deposition in the gas leads to a continuous spectrum down to the electronic noise level. Similar spectra are obtained by other types of counters like for helium-3 conversion [51], muons [52] or gammas [53].

**Figure 5:**
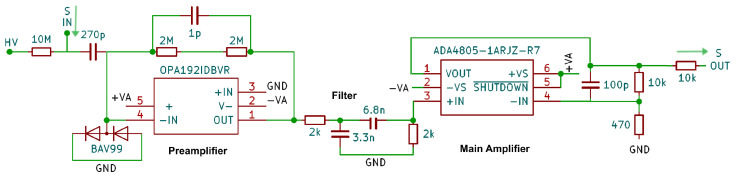
Input stage of the proportional counter readout. The signal is decoupled from the high voltage and fed into an integrating preamplifier. An optional band pass filter shapes the pulses before they will be scaled to the designated height in the main amplifier.

An onboard low-drift high-voltage module generates the bias for the anode [54]. This high voltage is DC-decoupled using a capacitor transmissible for high frequencies, as shown in Figure 5, where in the top left the counter tube is connected. The input stage starts with an integrating preamplifier that accumulates the charge signal of the detector. The product τ=RC of the resistor R=4MΩ and capacitor C=1pF determines the decay constant of its exponentially shaped output pulse, while its height *U* is determined by U=Q/C. Due to the small amount of generated charge *Q*, the pulses are rather low. The optimization process for τ mainly depends on the ion drift time scale, determined by the tube voltage and gas composition and pressure. τ needs to be large enough to integrate over the majority of the charge pulse, but in order to decrease the dead time, it should be integrated only as long as necessary. For our application, τ=4.4 µs was found to be appropriate. The following tight low- and high-pass filters were adapted to the typical frequency spectrum of the pulses with its cutoff frequencies $f_{C}=1/\left(2\pi RC\right)$ of 24 kHz and 12 kHz.

**Figure 6:**
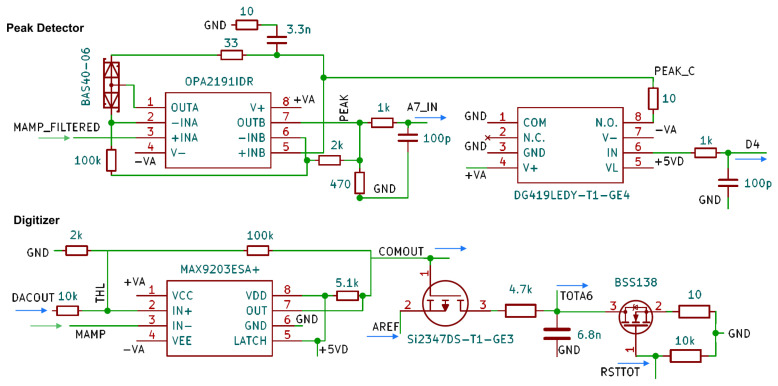
Sample and hold circuit (**top**) and pulse length measurement circuit (**bottom**). From the main amplifier, signals are fed in at the entry point ’MAMP’. The peak detector is read out on the A7 pin, while the comparator is connected to the COMOUT pin of the Arduino.

The analog output signal is then split; see Figure 6. One branch is forwarded to the comparator that triggers an output if the analog signal exceeds or falls below a threshold set by a digital-to-analog converter (DAC).

**Figure 7:**
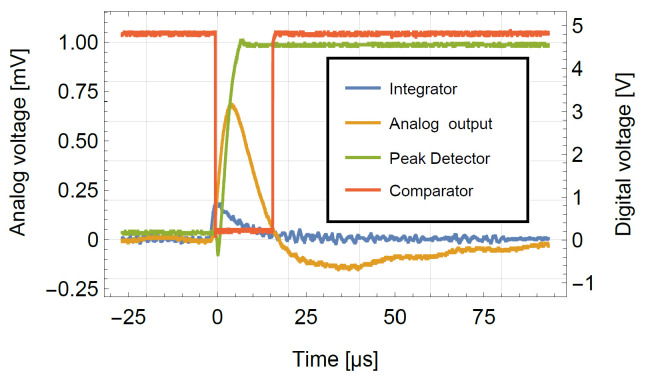
Signal shaping and working principle of the front-end electronics for typical pulse signals with the four stages. The signal amplitudes of the analog voltages (blue, orange and green curves) are shown on the left *y*-axis, and the digital levels of the signal, on the right *y*-axis (red curve).

The time difference between the falling and rising edge is recorded by the Input Capture Unit of the Arduino. The comparator output serves as a start and stop trigger for the pulse length measurement. The falling edge also triggers the readout of the analog signal through the 10-bit successive approximation register ADC of the Arduino. As the microcontroller requires a significant time for setting up and completing this voltage measurement, in order to ensure that the maximum of the pulse is sampled independently from the timing, a peak detector circuit stage follows the amplitude of the signal and holds its largest value [55]. The peak detector is reset after the analog conversion process is completed using a digital switch. Figure 7 shows the characteristic signal shapes after different stages on the board.

### 3.3. Photon Counter Readout

Contrary to ionization in solids and gases organic scintillating materials generate photons from decays of excited states to ground state vibration states. Such organic molecules [56] can be plastic [57] compounds that offer fast responses and low afterglow. Inorganic materials [58] are preferred for for their better energy resolution. Historically, scintillators have been read out by photomultipliers. With the rise of silicon photomultipliers [59] (SiPMs), their smaller detection surface required a downsizing of the active area. SiPMs are cost-effective avalanche photodiode arrays, which are operated slightly above their breakdown voltage. A single photon that interacts with the depletion zone can generate a free electron, which is accelerated by the electric field and consecutively releases more charge carriers (so-called Geiger mode). This process leads to the diode in one of the cells of the array becoming conductive in reverse direction. As soon as the avalanche stops, the diode is quenched to its original blocking state. The voltage drop over a short period by each interacting photon is measured as an inverted pulse.

**Figure 8:**
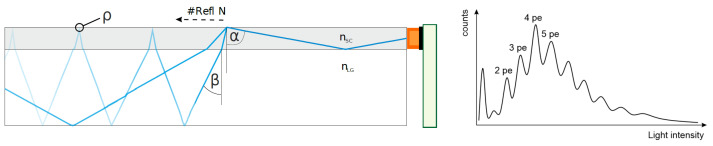
Working principle of a photon detector: A scintillator (white box) produces light from particles that pass through. A wavelength-shifting fiber (gray) with a different refractive index $n_{\mathrm{SC}}$ than the scintillator $n_{\mathrm{LG}}$ acts as an optical transmitter by reflecting (ρ) light of suitable angles α (translated from angles β inside the scintillator). A silicon photomultiplier (orange) records the intensity, which then yields a spectrum of pulses of simultaneously arriving photons (pe).

The size of such an SiPM lies in the order of millimeters, and the optical phase space of bulky scintillators cannot be compressed directly. Therefore, solutions are required that shrink the light output of the active material by a factor of 100. This is achieved using wavelength-shifting fibers [60], which are directly attached to the scintillator as shown in Figure 8. They translate the energy of the photon, and due to the different refractive index of the optical materials, some of the photons can be guided efficiently through the fiber. The SiPM mounted at its end generates pulses for which the signal height linearly translates to the amount of photons arriving at the same time. Depending on the total amount of energy deposited in the scintillator, one yields a spectrum, as shown in Figure 8 on the right panel. With the considerably poor resolution of plastic scintillators, typically, only coarse thresholds for signal ranges are required. Silicon photomultipliers have, however, the drawback that typical thermal energies at room temperatures can already free charge carriers, which leads to so-called dark noise in the order of 100 kHz. An effective method for suppressing this background is the coincidence counting of at least two detectors. As the probability of a randomly generated signal of a few 10 ns occurring at the same time is very low, this background is filtered out against synchronously arriving photons from a particle interaction. Further unwanted interferences of SiPMs are after-pulses and cross-talk, which both increase the amount of supposedly detected photons and hence impair operating the diodes at too high overvoltages.

We present two designs of readout boards with slightly different signal characteristics and different application focus. Both feature an amplifier, comparator and coincidence unit. The single-board v1 is designed to be directly attached to the scintillator. In order to keep a slim size, all voltages need to be fed in externally. The split-board v2 has a separate analog and digital stage that can be separated by a long cable. The tiny breakout board is equipped with the SiPMs on one side and an amplifier on the back side. Contrary to v1 the digital section of v2 generates all power levels including the SiPM supply and high voltage onboard. Our publication focuses on use cases for scintillators with wavelength-shifting fiber readouts. Although Silicon Photomultipliers can also be attached directly, the advantage of using WLSFs is that for one signal channel only two SiPMs are required. This aligns well with the capabilities of the Arduino microcontrollers. With an increasing number of individual SiPMs in place our concept becomes less efficient than more sophisticated readout systems. The systems are operated with SiPMs from Hamamatsu and AdvanSiD (see Table A1), and for an overview of the characteristics, see [61] or [62].

### 3.4. SiPM Single-Board SiPMTrigger v1

**Figure 9:**
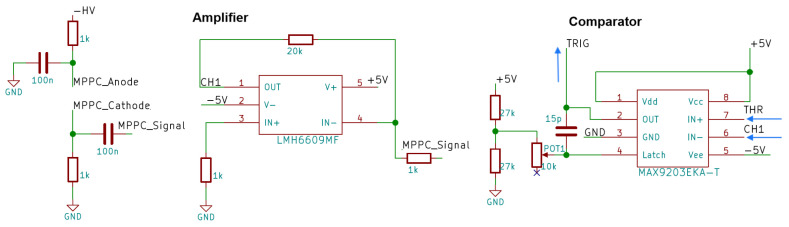
Schematic of the analog signal part of one channel of the single SiPM board v1 with the main components, from the SiPM to the digital trigger signal (blue arrow).

The single-board v1 is connected to the SiPMs by short cables. It feeds through the high-voltage supply, which needs to be fed in externally, and amplifies the photon pulses, which are then translated to TTL signals. Figure 9 shows the most relevant functional units of one channel. The high voltage is applied to the SiPM (also called Multi-Pixel Photon Counter (MPPC)) through resistors, which limit the maximum current in case of a breakdown and therefore serve as passive quenchers. With the SiPM being reverse biased at around 50 V, the signal needs to be DC-decoupled by a capacitor that is transparent for frequencies typical for the pulse. The inverting amplifier is configured for a gain of 20. The typical signal length is in the order of 100 ns and has a pulse height of several 100 mV after amplification. A latching comparator serves as a trigger, which outputs a TTL pulse with adjustable length. Both channels are led to a NOR gate (negated logical OR). This coincidence of both channels can be read out by the Arduino microcontroller on its interrupt pin.

### 3.5. SiPM Split-Board SiPMTrigger v2

**Figure 10:**
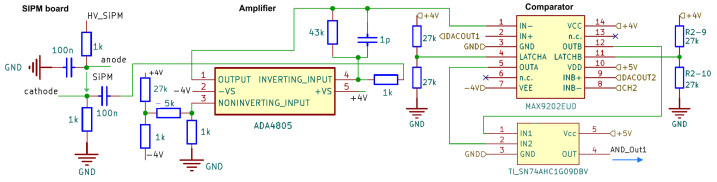
Schematic of the analog signal part of one channel of the split SiPM board v2. Compared to that in the board v1 in Figure 9, the signal polarity is inverted, and a dual channel comparator is used.

The SiPMTrigger board v2, shown in Figure 10, has a slightly different layout than the single-board v1 for the signal lines. The complete analog amplification stage with the SiPM is placed on a separate small-sized board that can be mounted directly onto the scintillator. The long cables to the SiPM are likely to increase the noise significantly, but with an already amplified output signal, this issue can be easily mitigated. With the possibility of keeping the digital stage separate from the detector, it is expedient to include all necessary power and allow the system to be supplied by 5 V DC only. Our system supports the power supply CAEN A7585D [63], or, optionally, Hamamatsu C11204-01 [64].

### 3.6. Data Logger

**Figure 11:**
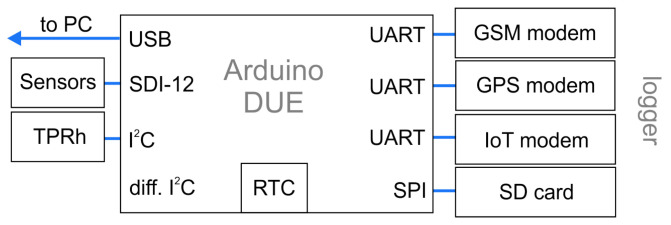
Main functional components of the Arduino-based data logger with the respective interfaces and protocols.

Data loggers are widely used in various applications, from laboratory data taking to environmental monitoring or process control. They allow data to be recorded over a period of time, enabling the detailed analysis and processing of the data. The previously presented front-end boards can be connected to a computer via USB. For data storage and slow control, it is also possible to build an independently operating data logger based on a 32-bit microcontroller like the Arduino DUE. Our approach, shown in Figure 11, utilizes the modularity and affordability of Arduino microcontrollers to develop a cost-effective data logger. By integrating modems for telemetry, data from different sources can be collected and transmitted wirelessly to a central server, increasing the flexibility and scalability of the system. The monitoring is completed using a time series data base like InfluxDB in combination with a graphical user interface like Grafana; a possible representation can be found in [65].

**Figure 12:**
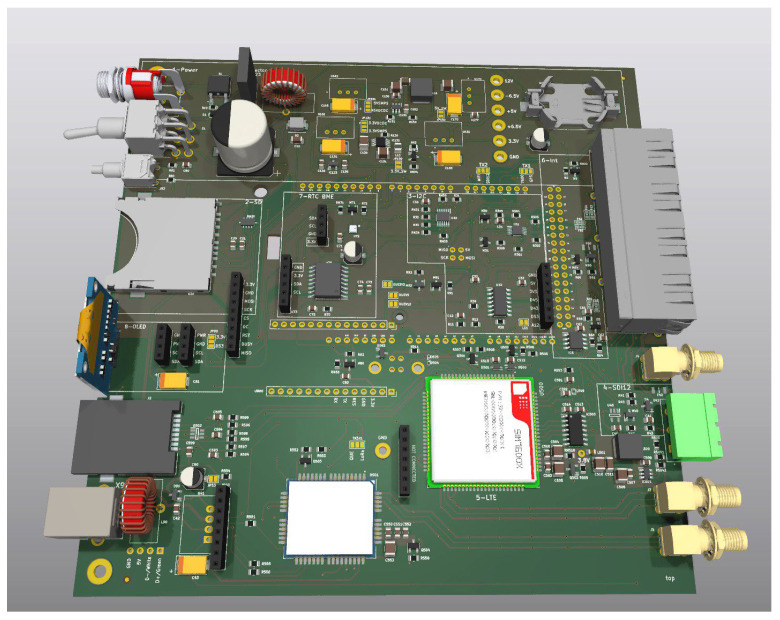
Three-dimensional rendering of the logger board. The Arduino DUE is inserted into the socket rows in the central part of the board. In the top segment, all voltages are generated from the 12 V DC-in connected to the power switch (top left). On the right side of the board, cable-mounted devices can be attached like sensors (four RJ45 ports, green SDI-12 sockets) and antennas (GPS and GSM). The left side is dedicated to periphery devices (from top to bottom) like the SD card, OLED display, SIM card and USB interface. Depending on the application, one of two modems (lower segment) can be operated, either with focus on the NB-IoT or LTE/GPS interfaces. The required antennas need to be connected to their respective SMA sockets.

Our data logger, see Figure 12, comprises the following components.

Energy-efficient voltage regulators: Choosing the most suitable powering scheme minimizes the energy consumption of the data logger, which is particularly important when the system is operated autonomously for long periods of time or used in applications with limited energy sources. We implemented switching regulators with a low quiescent current consumption and optimized the circuit topology for lower power loss. From an input of 12 V DC, adapted to the output voltage of most batteries and necessary for some sensors, the logger generates 5 V ($I^{2}$C, SPI), 3.3 V ($I^{2}$C, DUE) and 3.8 V (Modem). A step-up converter allows to generate 12 V for powering the system through a 5 V USB line.
Real-time clock and GPS: Our Arduino-based data logger has integrated temperature-compensated real-time clock (RTC) [66] and GPS [67] functionalities to provide accurate time stamps and geographical coordinates for the collected data. The RTC ensures a precise time synchronization of the data collection. With an integrated GPS, the data logger can automatically determine its geographical position, which is particularly important in applications with mobile or distributed sensor networks. The GPS also further improves the data accuracy by automatically readjusting the logger for small drifts in the RTC by synchronizing with the global GPS clock.
Storage: Data are saved on an SD card in the ASCII format in predefined intervals. The use of (industrial-grade, single-level-cell) SD cards as a storage medium enhances the flexibility of our data logger by allowing users to easily transfer to and analyze data on their computers without the need for special software or interfaces. In addition, continuous data recording, even in the event of an interrupted power supply or other faults, ensures the integrity of the collected data. The SD card also keeps the configuration file for the logger, which is loaded at startup for setting up the system without the need of reflashing the microcontroller.
Sensors: Our data logger is equipped with an integrated BME280 sensor [68] that measures temperature, humidity and air pressure. This sensor provides the precise detection of environmental conditions and enables the comprehensive monitoring of the environment, which is particularly important in weather applications. In addition, the data logger supports connection to SDI-12 sensors. The one-wire SDI-12 protocol [69] is translated to a UART port. SDI-12 is a widely used standard protocol for communication with environmental sensors like soil moisture and meteorological sensors.
Display: The one-inch LED display allows users to view important information, such as the system initialization at the bootup stage, measured values, status messages and configuration options, directly on the instrument without having to rely on external devices. This is particularly useful when commissioning and troubleshooting the system.
Telemetry: The data logger can be equipped with various communication modules, including the NB-IoT (Narrowband Internet of Things) [70] modem BC95-G [71], the 2G/4G modem Simcom SIM7600 [72] and the MKRWAN1310 [73] LoRa [74] modem. These modules enable wireless connectivity over various networks for remote data transmission. The BC95-G modem provides a connection for UDP (User Datagram Protocol) data through the NB-IoT network, which is specially optimized for transmitting small amounts of data over long distances with a low power consumption (LPWAN [75]). This functionality is ideal for sensors for which data integrity is not paramount and where tailored backends can be set up to receive the data. The SIM7600 module enables connectivity over 2G/4G networks, providing a fast internet connection for real-time data transfer, particularly via MQTT (Message queue telemetry transport) [76]. Besides HTTP (Hypertext Transfer Protocol) transport solutions, the transfer of data and log files using the FTP (File Transfer Protocol) is particularly useful for simpler and direct access. The MKRWAN 1310 provides LoRa communication, which enables low-power wireless communication over long distances, specifically in arrays of sensors distributed over large areas. Using this modem platform, the user can access an already established and widespread network of LoRaWAN gateways, facilitating the implementation of IoT applications. In the countryside, the connection range can be up to a few kilometers, whereas in urban areas, a few hundred meters can be achieved [77].

## 4. Results

### 4.1. Proportional Counter Readout

**Figure 13:**
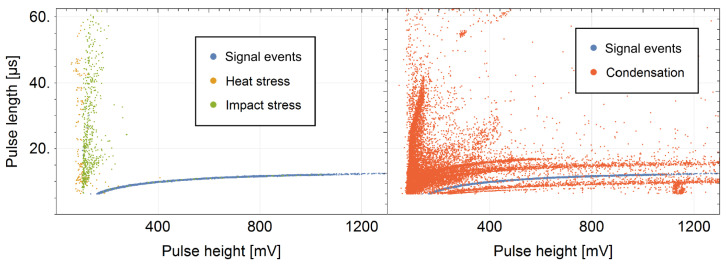
Pulse length–pulse height plot with induced noise aside the main signal events (blue). (**Left**): Heat (yellow) and mechanical stress (green) induce long but low pulses. (**Right**): The condensation of water vapor on the board (red) leads to a broad distribution of pulse shapes, which might originate from discharges as well as unwanted conductivity over humid surfaces.

The nCatcher was designed to address the requirements of measuring signals of counting tubes. A crucial aspect lies in precisely measuring the charge generated during ionization processes, distinguishing between particle types and suppressing unwanted events effectively. Leveraging well-adapted analog amplifier stages enables discrimination between dense and weak ionization tracks, which is crucial for particle identification. Additionally, the design emphasizes the precise determination of the charge pulse height and rise time, which are essential for accurate particle detection. Figure 13 shows examples that cover typical pulse-shape structures appearing under certain conditions, which have been amplified beyond usual use cases. The production of noise through heating the PCB to above 100 °C within a few minutes, mechanical stress induced by continuous impacts and the exposition of the electronics to 100% relative humidity can be distinctively discriminated against.

**Figure 14:**
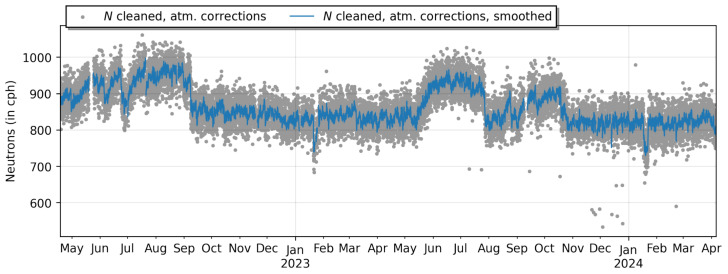
Time series of a neutron counter located in Mannheim, Germany, at 49.51471, 8.55178 over the period of two years. The system was exposed to usual environmental conditions ranging from −10 °C to 40 °C, including storms, heavy rain and lighting. Most of the points far below the average data originated from brownouts at low battery voltages.

Furthermore, the nCatcher addresses challenges related to temperature drifts, air humidity and background noise to ensure a reliable and robust performance in varied environmental conditions. The nCatcher was employed to measure neutron events in the frame of Cosmic Ray Neutron Sensing [78], in Mannheim, Germany, spanning a duration of two years; see Figure 14.

Throughout this extended period, the system operated under typical environmental conditions prevalent in the region, reflecting the real-world challenges encountered in outdoor monitoring scenarios. Despite these circumstances, the nCatcher consistently maintained its functionality and reliability, capturing neutron count data with precision and accuracy throughout the entirety of the observation period. Within the ADAPTER project [79], which spanned a comparable time range, more than ten stations were equipped with the nCatcher, and these have delivered data since their installation [80].

### 4.2. Photon Counter Readout

**Figure 15:**
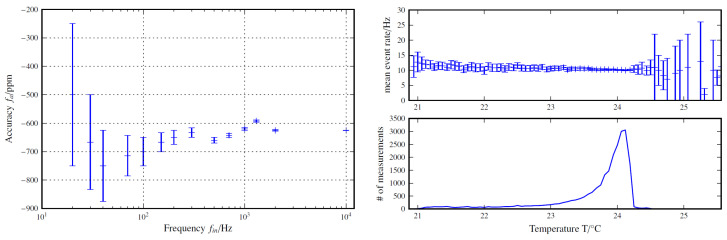
(**Left**): Accuracy of the frequency measurement of the SiPMTrigger board connected to the interrupt pin of the Arduino. (**Right**): Temperature stability test of the threshold adjustment for the SiPM spectrum.

To validate the consistency between the measured rate using the Arduino Nano and the trigger rate of the input signal, rectangular signals with various frequencies were generated utilizing a HP 33120A function generator and measured using the Arduino’s interrupt-capable digital input; see Figure 15 (left). For the accuracy of the SiPMTrigger, it was found that within the observed range, the measured frequencies were consistently less than 1%, deviating from the input frequencies and confirming the suitability for a frequency measurement within a small-scale experiment.

The amplification and signal amplitude of an SiPM exhibit a negative temperature coefficient, for which small temperature changes can already significantly alter the voltage levels. Consequently, a rise in temperature shifts trigger thresholds, affecting the measured trigger rates accordingly. To investigate this effect, trigger rates and temperature were measured over an extended period. Despite temperature variations, initial observations suggest no immediate change in trigger rates; see Figure 15 (right).

**Figure 16:**
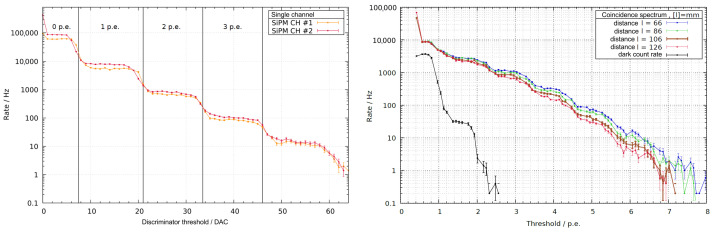
(**Left**): Dark count rate of two Hamamatsu S13360-1375PE SiPMs. (**Right**): Coincidence counting WLSF setup with a plastic scintillator with an alpha source placed at different distances from the readout.

The dark count rate of a silicon photomultiplier exhibits a profile of pulse heights in steps, which yields the typical background noise spectrum, as shown in Figure 16 (left). In a logarithmic plot, it is characterized by distinct plateaus. Each SiPM, composed of 285 parallel avalanche photodiodes (APDs), generates current pulses from activated APDs, resulting in quantized signal heights (pe), corresponding to the number of APD breakdowns within an event. This spectrum is fitted and used for the calibration of the threshold setting of the DAC. The smearing-out of the plateau, thereby, mostly originates from the buildup of the SiPM. In using the coincidence technique, the dark noise is reduced by orders of magnitude, as shown in Figure 16 (right). In the analysis presented in this figure, an alpha source was moved over a scintillator in order to trace the signal loss by reflections inside the material and the WLSF. This approach demonstrates the effectiveness of the coincidence measurement on the board in mitigating dark noise, thereby enhancing the signal-to-noise ratio.

### 4.3. Data Logger

**Figure 17:**
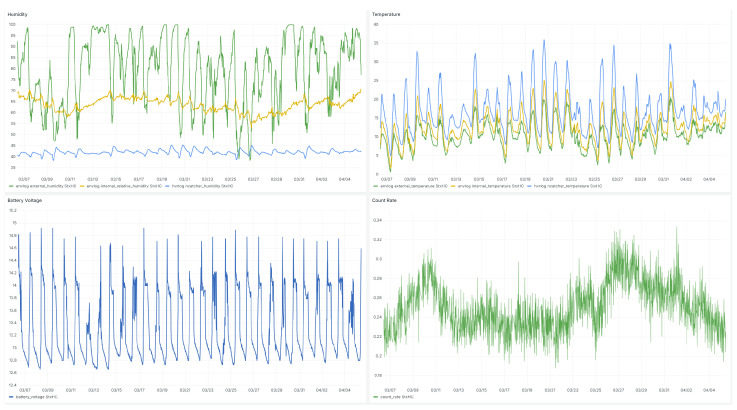
Example of the Grafana dashboard over a period of one month. The independently operating logger recorded data of environmental sensors like internal and external humidity, shown in the (**top-left**) panel; internal and external temperature, shown in the (**top-right**) panel; battery voltage, shown in the (**lower-left**) panel; and the count rate of a neutron detector attached to it, shown on the (**lower-right**) panel. All data were transmitted by a LoRa modem to a gateway and then sent via MQTT to the server, which stored it in a time series data base.

The data logger integrates various components to ensure efficient and reliable data acquisition across different applications with a focus on long-term monitoring under harsh conditions. It incorporates energy-efficient voltage regulators to minimize power consumption, which is essential for prolonged autonomous operation or applications with limited energy sources. Real-time clock and GPS functionalities provide accurate time stamps and geographical coordinates, enhancing the data precision, particularly in mobile or distributed sensor networks. Data storage on an SD card allows for easy data transfer and analysis, ensuring data integrity even during power interruptions. Equipped with a BME280 sensor and with support for SDI-12 sensors, the logger allows for environmental monitoring with a highly precise backup sensor. A one-inch LED display offers convenient onsite data visualization and system monitoring. Additionally, the logger can be equipped with various communication modules, including NB-IoT, 2G/4G, and LoRa for wireless data transmission over different networks. The results of such a data acquisition from the logger including all functionalities are exemplarily shown in Figure 17.

**Figure 18:**
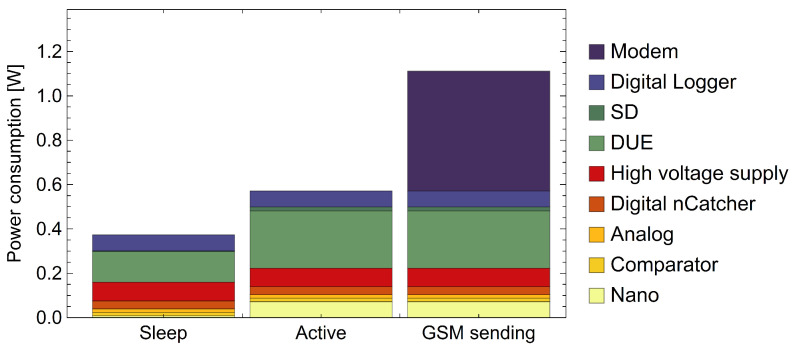
Power consumption of the data logger in operation combined with the proportional counter readout unit (nCatcher). Yellow to red color codes represent the nCatcher consumption, whereas green to blue color codes refer to the data logger components. The setup switches between three different modes. It predominantly resides in the sleep mode, which puts both microcontrollers and some parts of the digital domain in a stand-by mode. During the active stage, the data logger collects data from the nCatcher and peripheral SDI-12 devices and saves them on the SD card. According to user-defined intervals, the logger transmits data via 4G to a server.

The power consumption of a particle detector system comprising a data logger and nCatcher unit is depicted in Figure 18. The exact values change with the amount of proportional counters that are read out. Multi-counter systems will increase the consumption of the analog circuitry and the high-voltage power supply. The active stage, while the system acquires data, brings forth a higher power consumption of the digital circuitry, which includes the differential signal transmission between the logger and nCatcher, internal and external sensor readouts, and writing data on the SD card. With voltage regulators being more efficient at higher throughput currents at elevated power levels, the conversion efficiency is also increased. The average power demand depends on the time spent at each stage and, therefore, benefits from a long sleep-mode duration as well as large data transfer intervals. Mobile applications feature the constant use of a GPS positioning module, which adds significant current consumption to the overall budget.

## 5. Conclusions

The utilization of open hardware-based microcontrollers, particularly within the Arduino platform, has facilitated the rapid prototyping and implementation of innovative solutions. These devices, when coupled with specific front-end electronics, offer cost-effective alternatives for various applications ranging from student projects to small-scale instrumentation. The flexibility and extensibility of these platforms allow for the realization of various detector systems, as demonstrated through the presented examples involving proportional counter tubes and silicon photomultipliers. The SiPMTrigger and nCatcher designs show the adaptability of Arduino-based solutions for coincidence readout and pulse shape analysis. Serving different tasks in their distinct domains of requirements for count rate and signal precision, our solutions show the capabilities for stand-alone detector realizations in nuclear and particle physics. Researchers can benefit from the capabilities of our systems for precise signal detection, making them valuable tools for various experimental setups and applications. Additionally, the integration of a logger board enhances the data acquisition and slow control functionalities, thereby broadening the scope of applications for open hardware-based microcontrollers in research and education. For more advanced projects, the Arduino platform of the systems presented here can be exchanged for more professional microcontrollers that feature a broader range of functions and significantly better performance.

## Figures and Tables

**Table 1:** Summary of technical information about Arduino microcontrollers and STM32 examples for alternative counterparts for more advanced projects.

Type	Size	CPU	I/O	Volt-	Memory Size
	(mm)			age	
Arduino Nano	18 × 45	16 MHz	14	5 V	32 KB + 1 KB EEPROM
ATmega328					+ 2 KB RAM
Arduino DUE	102 × 53	84 MHz	54	3.3 V	512 KB ROM
AT91SAM3X8E					+ 96 KB RAM
Nucleo	18 × 45	80 MHz	(52)	3.3 V	128 KB ROM
STM32L412KBU [40]					+ 40 KB RAM
Nucleo	135 × 70	216 MHz	(168)	3.3 V	2 MB ROM
STM32F767ZIT6 [41]					+ 512 KB RAM

## Data Availability

Data and designs available on request from the authors. Further information can be obtained from the following project URLs: SiPMTrigger repository: https://gitlab.com/mkoehli/sipmTrigger (accessed on 29 April 2024), nCatcher repository: https://gitlab.com/mkoehli/ncatcher (accessed on 29 April 2024), Data logger repository: https://gitlab.com/mkoehli/arduinologger (accessed on 29 April 2024).

## Appendix A

The characteristics of the silicon photomultipliers, which were used in the context of this work, are summarized in Table A1.

**Table A1:** Electrical, optical and geometrical characteristics of the used silicon photomultipliers: peak sensitive wavelength λ, photo detection efficiency PDE at the peak wavelength, typical dark count rate $R_{D}$, gain factor *G*, breakdown voltage $U_{\mathrm{Br}}$, bias voltage $U_{\mathrm{Bias}}$, typical temperature coefficient at the recommended operating voltage ΔT, sensitive area $A_{S}$ and pixel pitch PP.

	Hamamatsu		AdvanSiD	
	S12571-050P	S13360-1375PE	ASD-RGB1S-P	ASD-NUV1S-P
λ	450	450 nm	550 nm	420 nm
PDE	35	50%	32.5%	43%
$R_{D}$	100 kHz	90 kHz	<100 kHz	<50 kHz
*G*	1.25 $\times \;10^{6}$	4 $\times \;10^{6}$	2.7 $\times \;10^{6}$	3.6 $\times \;10^{6}$
$U_{\mathrm{Br}}$	(65 ± 10) V	(53 ± 5) V	(27 ± 2) V	(26 ± 2) V
$U_{\mathrm{Bias}}$	$V_{\mathrm{Br}}+2.6$ V	$V_{\mathrm{Br}}+3$ V	$V_{\mathrm{Br}}+3$ V	$V_{\mathrm{Br}}+3$ V
ΔT	60 mV/°C	54 mV/°C	27 mV/°C	26 mV/°C
$A_{S}$	1 × 1 mm	1.3 × 1.3 mm	1 × 1 mm	1 × 1 mm
PP	75µm	75µm	40µm	40µm
